# Quantitative and Qualitative Approaches to Identifying Migration Chronology in a Continental Migrant

**DOI:** 10.1371/journal.pone.0075673

**Published:** 2013-10-09

**Authors:** William S. Beatty, Dylan C. Kesler, Elisabeth B. Webb, Andrew H. Raedeke, Luke W. Naylor, Dale D. Humburg

**Affiliations:** 1 Department of Fisheries and Wildlife Sciences, University of Missouri, Columbia, Missouri, United States of America; 2 Missouri Cooperative Fisheries and Wildlife Research Unit, United States Geological Survey, Department of Fisheries and Wildlife Sciences, University of Missouri, Columbia, Missouri, United States of America; 3 Missouri Department of Conservation, Columbia, Missouri, United States of America; 4 Arkansas Game and Fish Commission, Little Rock, Arkansas, United States of America; 5 Ducks Unlimited, Memphis, Tennessee, United States of America; Utrecht University, The Netherlands

## Abstract

The degree to which extrinsic factors influence migration chronology in North American waterfowl has not been quantified, particularly for dabbling ducks. Previous studies have examined waterfowl migration using various methods, however, quantitative approaches to define avian migration chronology over broad spatio-temporal scales are limited, and the implications for using different approaches have not been assessed. We used movement data from 19 female adult mallards (*Anas platyrhynchos*) equipped with solar-powered global positioning system satellite transmitters to evaluate two individual level approaches for quantifying migration chronology. The first approach defined migration based on individual movements among geopolitical boundaries (state, provincial, international), whereas the second method modeled net displacement as a function of time using nonlinear models. Differences in migration chronologies identified by each of the approaches were examined with analysis of variance. The geopolitical method identified mean autumn migration midpoints at 15 November 2010 and 13 November 2011, whereas the net displacement method identified midpoints at 15 November 2010 and 14 November 2011. The mean midpoints for spring migration were 3 April 2011 and 20 March 2012 using the geopolitical method and 31 March 2011 and 22 March 2012 using the net displacement method. The duration, initiation date, midpoint, and termination date for both autumn and spring migration did not differ between the two individual level approaches. Although we did not detect differences in migration parameters between the different approaches, the net displacement metric offers broad potential to address questions in movement ecology for migrating species. Ultimately, an objective definition of migration chronology will allow researchers to obtain a comprehensive understanding of the extrinsic factors that drive migration at the individual and population levels. As a result, targeted conservation plans can be developed to support planning for habitat management and evaluation of long-term climate effects.

## Introduction

Migration is a fundamental aspect of natural history in many species and has profound implications to vital rates and individual fitness [1], [2]. A variety of taxonomic groups exhibit some form of migration, including mammals [3], amphibians [4], insects [5], fish [6], and birds [7]. Although definitions of migration differ among taxa, the term generally represents individual movements that collectively facilitate a population level outcome [8]. Specifically in birds, migration is considered an annual event with distinct seasonal movements between breeding and non-breeding areas [9]. Consequently, energy and nutrient acquisition during migration can affect body condition, reproduction, and survival throughout the annual cycle [10]–[12].

North American waterfowl exhibit some of the most visible seasonal migrations, moving between breeding and wintering areas in the spring and autumn [13]. Waterfowl migration has not been thoroughly studied at the individual level and proximate factors that influence migration chronology remain unclear, especially among dabbling ducks [14]. In addition to a limited understanding of extrinsic migration drivers, waterfowl movement and seasonal ranges are increasingly affected by anthropogenic disturbances [15]–[17]. For example, climate change has the potential to dramatically alter spatial and temporal migration patterns in North American waterfowl [16], [17]. As a result, quantitative methods are required to adequately monitor the timing (initiation and termination dates) and duration of migration in waterfowl at the individual, population, and species levels.

Waterfowl migration has been examined using a variety of methods, including counts [18], radar [19], and very high frequency (VHF) radio-telemetry [20]. Despite these efforts to document migration chronology, past studies have been limited spatially (counts, VHF radio-telemetry), temporally (radio-telemetry, banding), or taxonomically (radar) by available technology. However, recent advances in satellite telemetry provide opportunities to monitor migrating species and individuals throughout the annual cycle at broad spatio-temporal scales [21], [22]. Although several approaches have been used to quantify migration chronology in waterfowl, a flexible and quantitative framework for examining life history chronology in individuals is lacking for medium- and long-distance avian migrants. Thus, the objective of our study was to quantify and compare the timing and duration of seasonal waterfowl migrations using different techniques. To achieve this objective, we tracked 19 female adult mallards (*Anas platyrhynchos*) equipped with global positioning system (GPS) satellite transmitters and used quantitative and qualitative approaches to define migration at the individual and population levels. At the individual level, we used a geopolitical method that has been previously used to evaluate waterfowl migration and movements among state, provincial, and international boundaries [23]–[25]. We also used a second individual approach that has been applied to short-distance migratory ungulates (i.e. <220 km) to model a distance-based metric as a function of time [26], [27]. At the population level, we modeled the distance-based metric in a mixed model framework that accounted for variance within and among individual birds.

## Methods

### Ethics Statement

Mallards were captured, banded, and marked with backpack GPS satellite transmitters in Arkansas, USA by Arkansas Game and Fish Commission personnel under United States federal banding permit 06569. All reasonable efforts were made by Arkansas Game and Fish Commission and Ducks Unlimited Canada field personnel to minimize animal suffering.

### Capture and GPS Telemetry

Adult mallard hens were captured and marked in two separate cohorts. The first cohort was captured using swim-in traps [28] near Yorkton, Saskatchewan (SK), Canada (51°13′N 102°28′E) in late September 2010. The second cohort was captured with rocket nets [29] in mid-February 2011 at multiple locations in Arkansas (AR), USA (Five-Oaks Duck Lodge at 34°20′N, 91°36′E; Bayou Meto Wildlife Management Area at 34°13′N, 91°31′E; Black River Wildlife Management Area at 36°03′N, 91°09′E). Captured individuals were outfitted with a Teflon ribbon harness [30] equipped with a solar-powered GPS satellite transmitter programmed to obtain four GPS locations per day (Model PTT-100, Microwave Telemetry, Inc., Columbia, Maryland, USA). GPS units were active long enough to obtain a location provided sufficient battery life. For birds captured in AR, the completed transmitter and harness (28 g) accounted for ≤3% of body mass ( = 1099.0 g, SD = 71.5), with the exception of two individuals that were fit with a larger transmitter model (38 g harness) that accounted for 3.8% and 3.3% of individual body mass, respectively [31]. Birds captured in SK were not weighed, but we speculate the harness accounted for ≤3% of body mass for all but 2 individuals outfitted with the larger transmitter. All marked birds were held for at least four hours to ensure individuals acclimated to harnesses before release. We monitored marked birds until transmitters failed or a transmitter was immobile for a period of time. We identified immobile transmitters by evaluating each GPS location based on the distance it was from the last recorded location for each individual. Specifically, locations ≤100 m from the last recorded location for each individual were removed from further analyses.

We used several criteria to determine whether an individual should be included in migration analyses. Ducks marked in SK in September 2010 with immobile or failed transmitters before 1 January 2011 were excluded from analyses to ensure the animals had the opportunity to complete the autumn migration. Similarly, for the February 2011 cohort marked in AR, ducks with immobile or failed transmitters before 1 June 2011 were excluded from analyses to ensure the animals had the chance to complete the spring migration. A subset of animals was tracked into a second year (>365 days) and similar criteria were applied to the second year of data (1 January 2012 or 1 June 2012). We specifically removed birds that did not have a chance to complete migration to prevent any biases in migration model parameter estimates (e.g. migration distance, timing of migration).

### Geopolitical Boundaries

We used two separate approaches to define the timing and duration of migration for each individual, and we applied a third method that defined migration for the population. The first approach employed geopolitical boundaries to approximate movement among relevant management units for migratory birds (e.g. countries, provinces, states). Initiation of autumn migration for the SK cohort was defined as the date that a bird was last located in SK and when the succeeding location was south of SK. The end of autumn migration was defined as the date that the marked bird reached the most southern state along the migration route. Likewise, for the AR cohort, initiation of spring migration was defined as the date that a marked duck was last located in AR with a subsequent location north of AR. The end of spring migration was defined as the date the marked bird arrived at the most northern state or province along the migration route and remained in the state or province for at least 30 days. We chose 30 days to approximate the incubation period for mallards [32] and to be consistent with previous studies that have analyzed the timing of migration in dabbling ducks [7], [23]–[25]. We calculated mean departure, midpoints, and arrival dates for each season and year to obtain population level estimates for the timing and duration of migration.

### Individual Net Displacement Models

**Figure 1:**
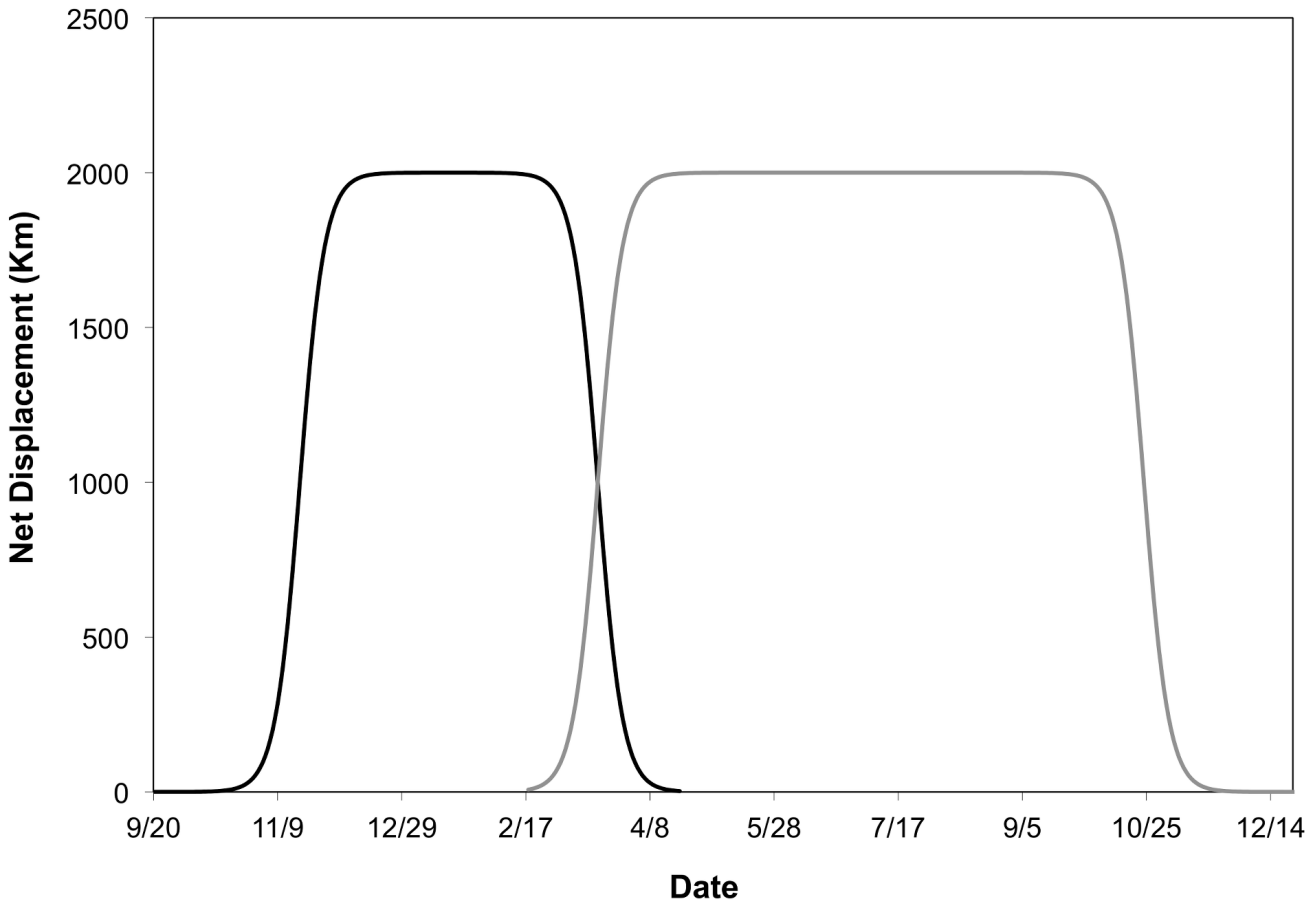
Predicted net displacement (ND) as a function of time for two idealized individuals. The black line represents a duck that was initially marked on the nesting grounds in Saskatchewan, Canada in September 2010. The gray line represents an individual that was marked on the wintering grounds in Arkansas, USA in February 2011.

The second approach that we used to define the timing and duration of migration was a quantitative method based on net displacement (ND). The ND measures the Euclidean distance between the initial location and each subsequent relocation for an individual [33], [34]. Consequently, ND is expected to vary as a function of season, and its interpretation depends on the timing and location of marking [26], [27]. Thus, ND patterns for birds marked on the wintering grounds would have different interpretations than birds marked on the nesting grounds (Figure 1). Specifically, for birds marked in AR (wintering grounds), individuals would exhibit increased ND with the onset of the spring migration and an eventual stabilization near a maximum value once the individual had reached the nesting grounds (Figure 1). At the onset of the autumn migration, ND would decrease and approach zero as birds neared the wintering grounds. For birds captured on the nesting grounds in SK, ND would exhibit a similar trend in a migratory individual, although peak displacement values would occur during the winter instead of the summer (Figure 1).

Although net squared displacement (NSD) has been used to model animal movements, [26], [27], [33], [34], we chose to model ND because we were specifically interested in the initiation date, midpoint, termination date, and duration of migration rather than migration distance. In an NSD framework, the nonlinear nature of the squared transformation has the potential to temporally shift migration seasons compared to the ND metric because the squared term compresses distance values at the lower end of the scale and expands distance values at the higher end of the scale. As a result, migration from the origin would initiate and terminate later in an NSD framework compared to the ND scale.

To quantify temporal trends in ND, we fit two candidate nonlinear models to predict ND as a function of time for each individual [26], [27]. Data for individuals tracked >365 days were separated by year. We then iteratively fit two candidate models to each duck-year dataset based on the methods presented in [26]. A three-parameter logistic growth model (i.e. single-sigmoid) denoted an individual that was tracked for a single migration [26], [35]:
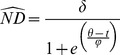
(1)where 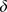 is the asymptotic height and the distance of migration, is the date at which migration reaches its midpoint at 50% of asymptotic height, *φ* is the time passed between the midpoint of migration 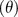 and approximately 73% of asymptotic height, and *t* is the number of days passed since the first recorded location near the capture site [26], [35]. We considered the migration period to begin at 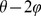 and terminate on 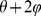 to correspond to approximately 12% and 88% of asymptotic height, respectively. We chose 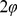 to delineate migration in dabbling ducks due to their propensity for extended stopover stays and to provide a more accurate temporal estimation of migration [26], [32].

We also fit a double-sigmoid function to each duck-year dataset to model full migration [26], [35]:
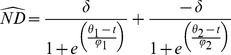
(2)


Parameters were similar to those from the single-sigmoid model (eqn 1), except that the double-sigmoid model contained separate estimates of the midpoint and timing of migration for autumn 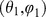 and spring 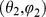 migrations [26].

Candidate models (eqn 1, eqn 2) were fitted using the base stats package in R (nls function) [36] and a plausible range of starting values was applied to obtain model convergence. Models were ranked according to Akaike’s information criterion adjusted for small sample size (AIC*_c_*) [37] and each duck-year dataset was categorized according to its marking cohort (AR or SK) and top model (single- or double-sigmoid). Thus, each duck-year was classified as AR-single, AR-double, SK-single, or SK-double using AIC*_c_*. If the top model contained ecologically implausible parameter estimates (e.g.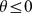), the duck-year was categorized with the alternative model. Parameter estimates were used to make inferences on the timing and duration of migratory events separately for each individual duck-year based on the top model. Although defining migration separately for each duck-year may overstate variation in migration patterns among individuals [38], we had a substantial sample of locations for each duck-year. Thus, we felt that models would provide valid inferences for the timing and duration of migration for each individual.

### Statistical Differences in Migration Parameters

We used analysis of variance (ANOVA) to ensure that migration parameter estimates for individual ND models were not biased between model categories (proc glm; SAS, Cary, North Carolina, USA). Four separate models examined the effects of individual model classification (single- or double-sigmoid) on migration (1) initiation date, (2) midpoint, (3) termination date, and (4) duration. We did not detect differences between model categories for any of the migration parameters examined (see results), therefore, further analyses included migration parameter estimates from both individual model categories (single- or double-sigmoid) unless otherwise noted.

We also used ANOVA to examine the effects of season (autumn or spring) and method (geopolitical or individual ND) on the duration of migration while controlling for variability between marking cohorts (AR or SK) and years (proc glm; SAS). Marking cohort (SK or AR), season (autumn or spring), and method (geopolitical or individual ND model) were denoted as independent class variables, and the duration of migration (number of days) was the dependent variable. Additionally, ANOVAs were used to evaluate the effects of method (geopolitical or individual ND) on the initiation date, midpoint, and termination date separately for spring and autumn migration. In these six models (three migration parameters × two migration seasons), we again included year and marking cohort as independent class variables. In all ANOVAs, residual plots were examined to ensure assumptions were met for linear models. Effect sizes were evaluated with the semipartial ***ω*** *^2^* statistic, which measured the proportion of total variance in the dependent variable attributed to the focal independent variable [39]. Additionally, Tukey’s post-hoc tests for multiple comparisons were used to evaluate differences between groups provided a significant overall test.

### Mixed-Effects Net Displacement Models

Mixed-effects models incorporate variance within and among groups (i.e. individuals) to estimate population level parameters [35], [38]. Thus, in our third approach, we inferred population level migratory patterns using mixed models that allowed the asymptote 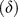 and timing 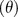 parameters to vary across ducks. We fit four separate mixed-effects models that corresponded to the aforementioned marking cohort/top model categories (AR-single, AR-double, SK-single, SK-double). Based on fixed effects parameter estimates, we calculated population level estimates for the timing and duration of migration. Mixed-effects models were fit in the nlme package [40] in R and a range of plausible starting values was applied to obtain model convergence. This method did not generate individual level estimates for migration parameters; rather it produced a population level estimate that accounted for variation within and among ducks.

## Results

### Capture and Telemetry

We captured and marked a total of 40 adult mallard hens over the course of the study (20 in SK, 20 in AR). However, seven ducks were lost shortly after release (five immobile transmitters, two failed transmitters) so our sample was reduced to 33 individuals. The mean number of locations per individual was 622.0 (range: 103.0–2544.0, SD = 550.7), and birds were tracked for an average of 216.8 days (range: 49.8–723.0, SD = 181.6). We defined timing and duration of migration for 19 ducks, four of which were tracked for two years. Thus, 23 duck-year datasets met the 1 January/1 June criteria for inclusion in analyses.

### Geopolitical Boundaries

**Figure 2:**
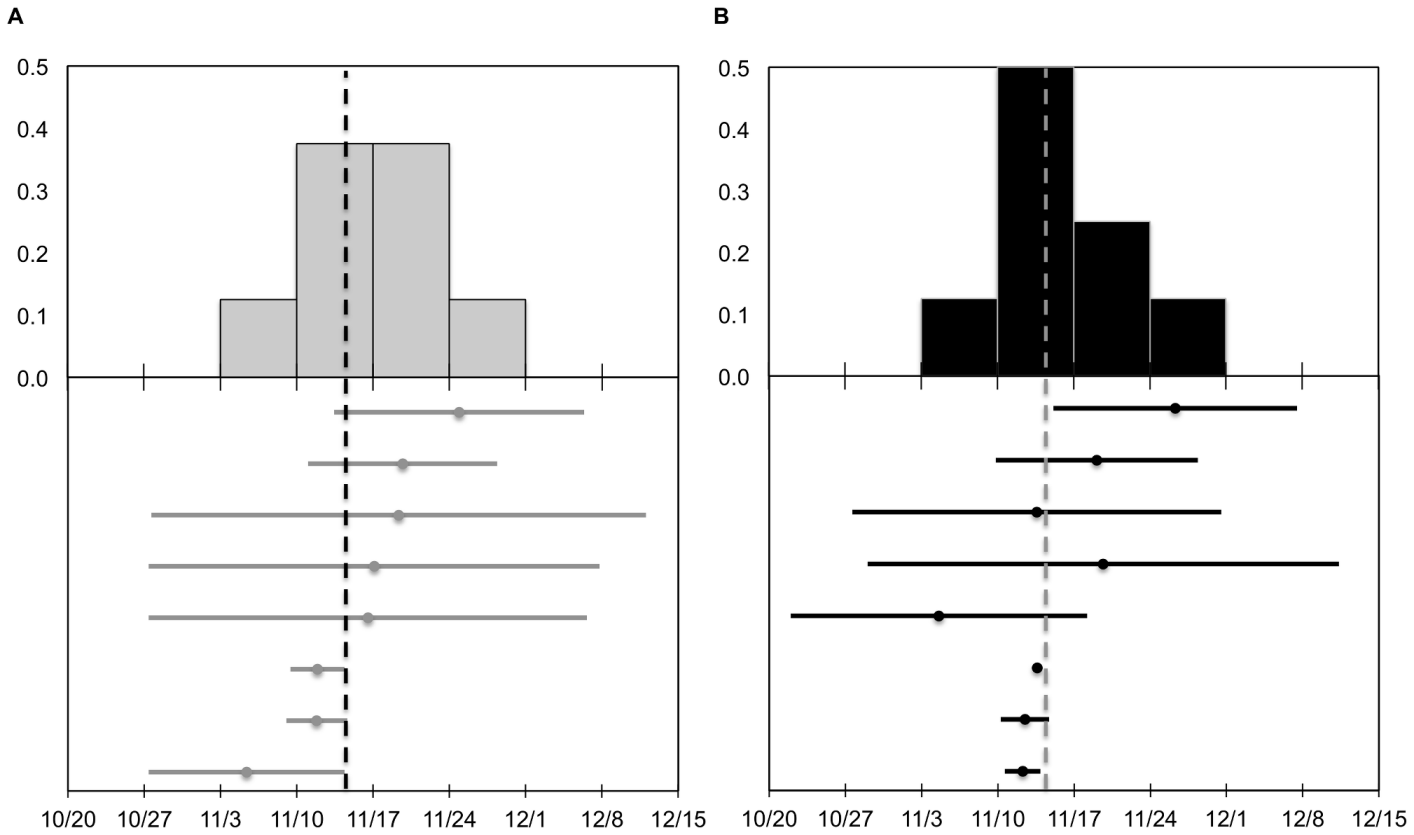
The midpoint and duration of migration in autumn 2010 for eight adult mallard hens. Upper panes illustrate the frequency distribution for the midpoint of autumn migration according to (a) geopolitical method and (b) ND approach. Lower panes illustrate corresponding migration midpoints (circles) and extents (bars) for each of the methods. Vertical dashed lines represent the mean migration midpoint.

**Figure 3:**
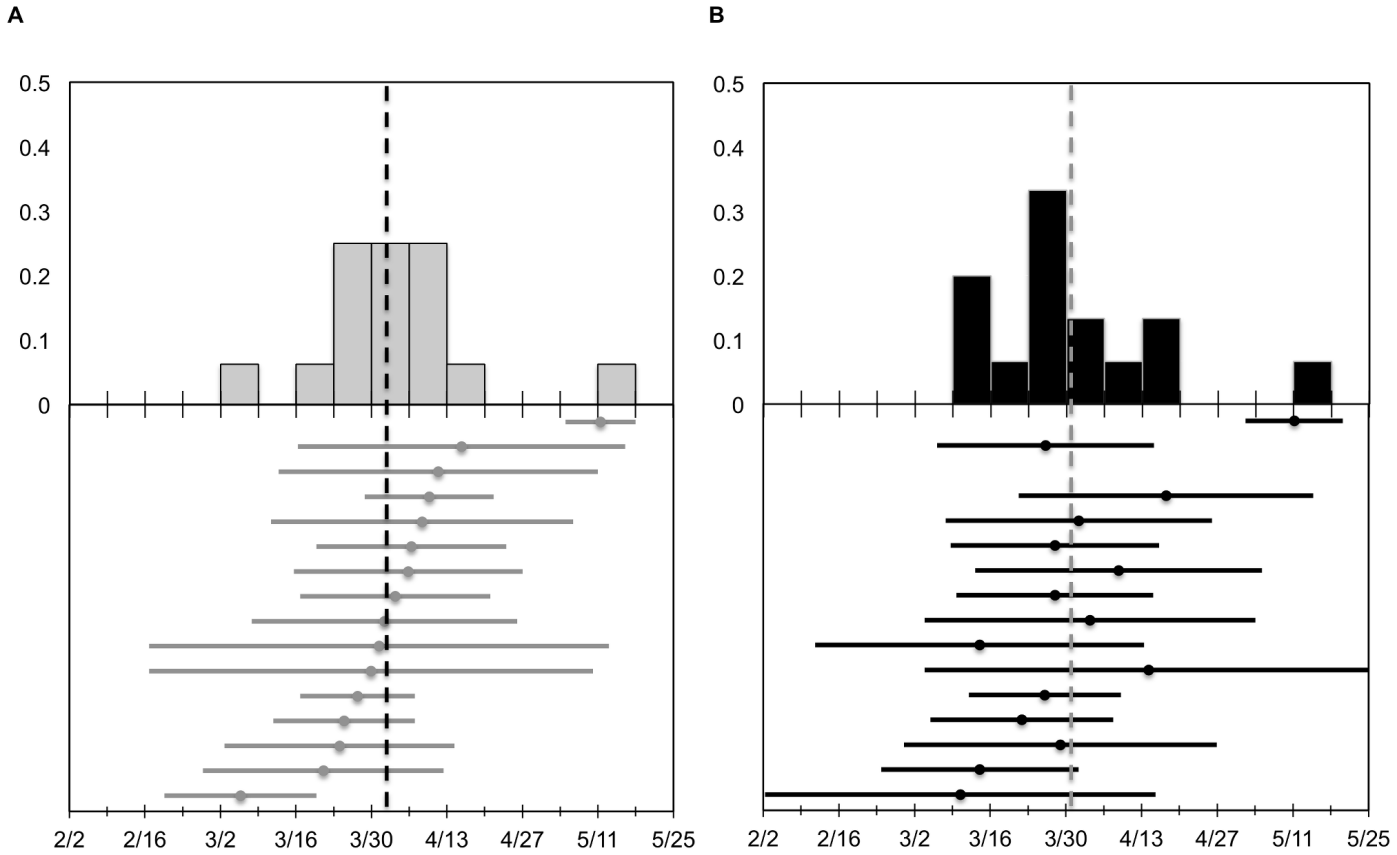
The midpoint and duration of migration in spring 2011 for 16 adult mallard hens. Upper panes illustrate the frequency distribution for the midpoint of spring migration according to (a) geopolitical method and (b) ND approach. Lower panes illustrate corresponding migration midpoints (circles) and extents (bars) for each of the methods. Vertical dashed lines represent the mean migration midpoint.

**Figure 4:**
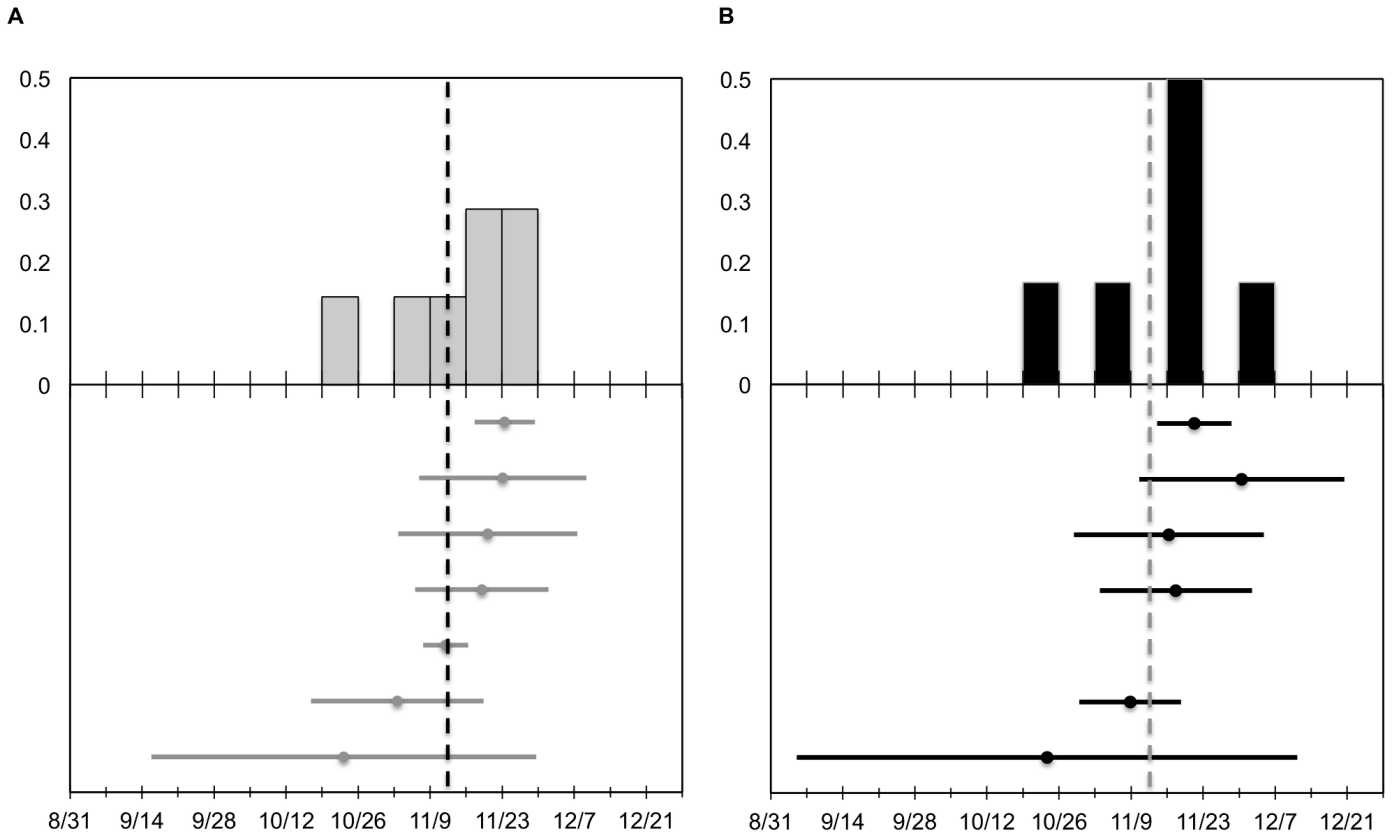
The midpoint and duration of migration in autumn 2011 for seven adult mallard hens. Upper panes illustrate the frequency distribution for the midpoint of autumn migration according to (a) geopolitical method and (b) ND approach. Lower panes illustrate corresponding migration midpoints (circles) and extents (bars) for each of the methods. Vertical dashed lines represent the mean migration midpoint. See text for a description of sample size discrepancy between (a) and (b).

Based on the geopolitical method for delineating migratory events, autumn migration in 2010 (*n* = 8) had a mean midpoint of 15 November 2010 (SD = 6.0 days) and mean duration of 24.5 days (SD = 16.1) (Figure 2a). Spring 2011 migration had a substantially larger sample size (*n* = 16) with a mean midpoint of 3 April 2011 (SD = 14.2) and mean duration of 44.1 days (SD = 20.8) (Figure 3a). Autumn 2011 migration (*n* = 7) initiated as early as 15 September 2011 and exhibited a mean midpoint of 13 November 2011 (SD = 11.8) with a mean duration of 31.7 days (SD = 21.7) (Figure 4a). Spring 2012 migration (*n* = 3) had a mean midpoint of 20 March 2012 (SD = 18.9) and mean duration of 26.3 days (SD = 17.0).

### Individual Net Displacement Models

Migration parameters derived from individual ND models were similar to those based on the geopolitical method. However, two particular duck-year datasets included data for approximately one year, yet were assigned the single-sigmoid model. Thus, for spring 2011 and autumn 2011 migrations, the geopolitical method contained one additional sample compared to the individual ND method (see below). Autumn migration in 2010 (*n* = 8) had a mean midpoint of 15 November 2010 (SD = 6.5) with an average duration of 19.2 days (SD = 15.6) (Figure 2b). For spring migration in 2011 (*n* = 15), the mean midpoint was 31 March 2011 (SD = 15.7) and average duration was 48.2 days (SD = 17.4) (Figure 3b). The mean midpoint for autumn migration in 2011 (*n* = 6) was 14 November 2011 (SD = 12.8) with an average duration of 39.6 days (SD = 29.8) (Figure 4b). Although the sample size for spring migration 2012 was relatively small (*n* = 3), the mean midpoint was 22 March 2012 (SD = 20.5) and the average duration was 47.7 days (SD = 31.2).

### Differences in Migration Parameters

The date of migration initiation (*F*_1, 30_ = 0.49, *p* = 0.49, *R^2^* = 0.02), migration midpoint (*F*_1, 30_ = 0.76, *p* = 0.39, *R^2^* = 0.02), migration termination (*F*_1, 30_ = 1.12, *p* = 0.30, *R^2^* = 0.04), and the duration of migration (*F*_1, 30_ = 1.96, *p* = 0.17, *R^2^* = 0.06) did not vary due to individual model classification (single- or double-sigmoid). Thus, all subsequent analyses pooled information from both individual ND model categories.

Although the duration of migration varied substantially (*F*_5, 60_ = 3.18, *p* = 0.01, *R^2^* = 0.21), the effects of method (*F*_1, 60_ = 0.69, *p* = 0.41, ***ω*** *^2^* = 0.00, ***ω*** *^2^* 95% CI = 0.00–0.10), migration season (*F*_1, 60_ = 2.49, *p* = 0.12, ***ω*** *^2^* = 0.02, ***ω*** *^2^* 95% CI = 0.00–0.15), marking cohort (*F*_1, 60_ = 0.03, *p* = 0.86, ***ω*** *^2^* =  -0.01, ***ω*** *^2^* 95% CI = 0.00 to 0.05), and year (*F*_2, 60_ = 1.57, *p* = 0.22, ***ω*** *^2^* = 0.01, ***ω*** *^2^* 95% CI = 0.00–0.15) did not have a substantial influence on migration duration. Additionally, the initiation date (*F*_3, 25_ = 1.50, *p* = 0.24, *R^2^* = 0.15), midpoint (*F*_3, 25_ = 2.16, *p* = 0.12, *R^2^* = 0.21), and termination date (*F*_3, 25_ = 1.45, *p* = 0.25, *R^2^* = 0.15) of autumn migration did not vary according to method, marking cohort, or year. Similarly, for spring migration the initiation date (*F*_3, 33_ = 0.49, *p* = 0.69, *R^2^* = 0.04), midpoint (*F*_3, 33_ = 0.84, *p* = 0.48, *R^2^* = 0.07), and termination date (*F*_3, 33_ = 1.06, *p* = 0.38, *R^2^* = 0.09) did not vary as a function of method, marking cohort, or year (Table S1). Thus, the specific methodological approach used to define migration did not substantially affect the estimated duration, initiation date, midpoint, or termination date of spring or autumn migrations.

### Mixed-Effects Net Displacement Models

**Figure 5:**
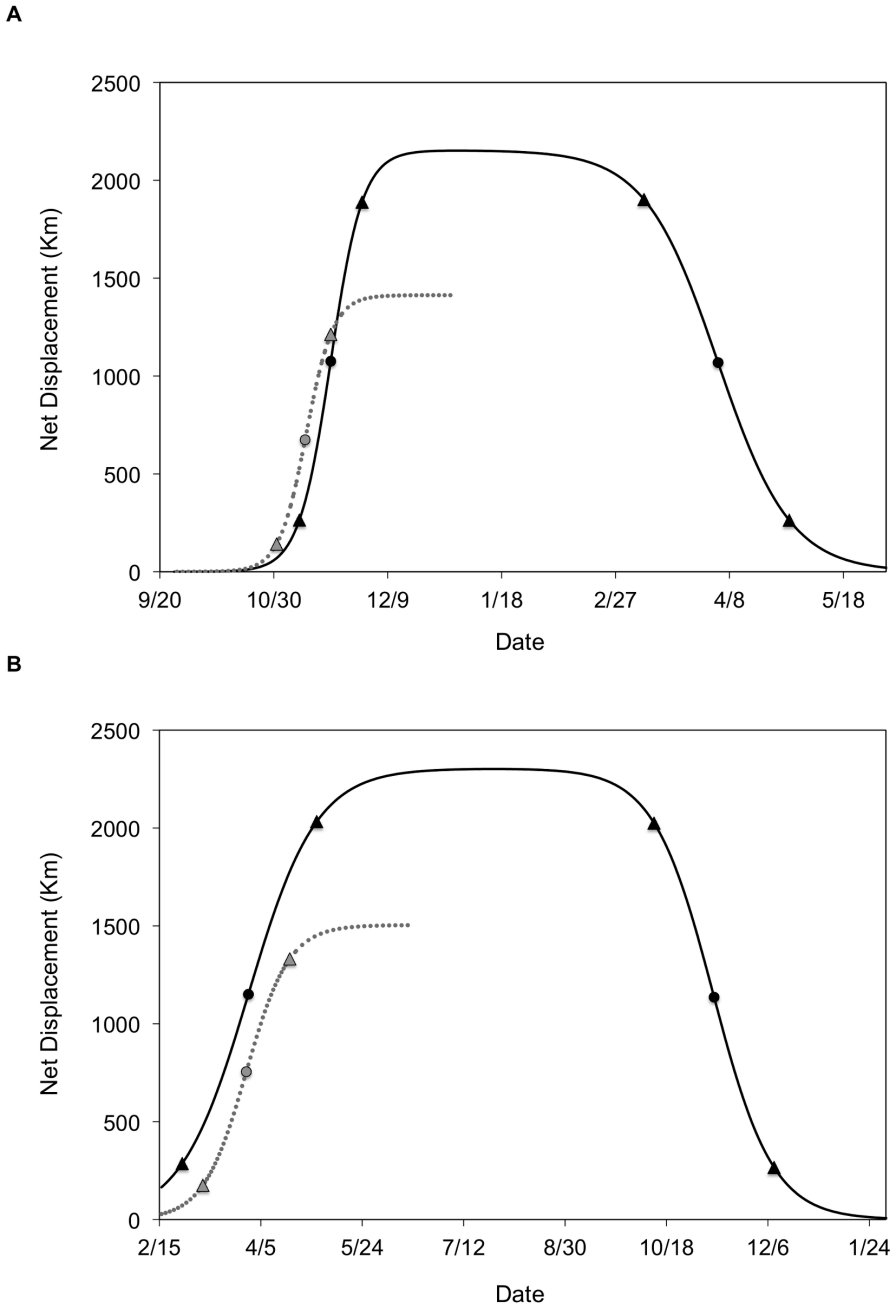
Nonlinear mixed-effects migration models for ducks marked in Saskatchewan, Canada (top pane) and Arkansas, USA (lower pane). Predicted net displacement is displayed on the y-axis and time is on the x-axis. Black lines represent predicted ND values over time for the double-sigmoid model and gray lines represent predicted values for the single-sigmoid model. The population level midpoints (circles) and initiation and termination of migration are also displayed (triangles) for single- and double-sigmoid models.

Mixed-effects models contained random effects terms for the asymptote and midpoint parameters and only included data from the first year of tracking as small sample sizes precluded separate analyses of data from the second year (Figure 5). Based on these population models, the autumn 2010 migration midpoint was 10 November 2010 for the single-sigmoid model and 19 November 2010 for the double-sigmoid model, with duration estimates of 19.0 and 22.4 days, respectively (Table S2). For spring migration in the SK cohort, we estimated a migration midpoint of 4 April 2011 and duration of 51.1 days for the double-sigmoid model. The midpoint of spring migration for the AR cohort was 29 March 2011 (41.3 day duration) and 30 March 2011 (65.6 day duration) for the single- and double-sigmoid models, respectively (Table S2). For autumn 2011 migration, the AR cohort exhibited a midpoint of 10 November 2011 and average duration of 57.8 days for the double-sigmoid models (Figure 5).

## Discussion

Information about migration is critical to better understand waterfowl ecology and help inform local, regional, national, and international conservation planning [41]. Despite the importance of migration within the annual cycle and the associated conservation and management implications, quantitative techniques that define waterfowl migration chronology are rarely used. We compared individual migration chronology estimates from a quantitative technique based on a distance metric (ND) with estimates from a geopolitical method that examined bird movement relative to state, provincial, and international boundaries [23]–[25]. We did not detect meaningful differences between the methods with regard to migration initiation, midpoint, termination, or duration in the autumn or spring.

In addition to the individual ND and geopolitical methods, we used a mixed model that accounted for variation among individual birds to provide a population level assessment of migration chronology. Random effects to address variation among years and populations could be included to obtain a more comprehensive model of migration chronology, although adequate sample sizes would be required [26], [27]. Additionally, mixed models based on ND could be evaluated in the context of local, regional, and continental surveys that focus on population level estimates. For example, observation networks (e.g. the Mallard Migration Observation Network, Integrated Waterbird Management and Monitoring Program) provide information on the chronology of migration at broad spatial and temporal scales. With these approaches, a network of environmental managers monitors the progression of migration over time to provide a continental scale interpretation of migration chronology by season. At the local scale, duck abundance could be monitored on multiple wetlands over time to document migration chronology and compared to chronologies derived from tracking data [18].

Although differences in migration chronology were not evident between the individual ND and geopolitical methods, there are several advantages to the ND method. Specifically, ND provides a framework for further development of migration models in migrating birds and has the potential to model more complex migration behaviors. For example, stopover areas are considered ecologically important habitats to migrating waterfowl, yet research on the importance of specific stopover areas is lacking [14]. Change point models developed within an ND framework could provide a quantitative approach to defining prominent stopover areas, although frequentist techniques used to estimate regression models with an unknown number of change points are limited to continuous regression lines [42]. However, Bayesian change point models have been the focus of recent research and allow simultaneous estimation of the number and location of breakpoints as parameters in discontinuous regression lines [43].

Despite the empirical and theoretical advantages to using ND, an emphasis on interpreting temporal trends in ND within the context of the annual cycle events that occur at the capture site is warranted. For example, birds captured at a migration stopover site would exhibit different temporal trends in ND compared to birds captured on the wintering or nesting grounds. In addition, models applied in this study were ideal for philopatric individuals that return to the same geographical area each year [26]. A second asymptote could be included in the model (eqn 2) to account for individuals, populations, or species that did not exhibit fidelity to nesting or wintering areas [*sensu* 26]. However, Bayesian change point models that identify the number and location of change points would provide the most flexible modeling approach to ND [43].

Displacement methods (i.e. NSD) were initially validated using simulated and empirical data from ungulates that migrate over relatively short distances [26], [27], yet are sufficiently flexible to be applicable to data collected from a variety of species, devices, and movement distances provided marked individuals represent an unbiased sample of the population. Although quantifying migration requires telemetry data from a population of individuals tracked at a broad spatio-temporal scale, high-resolution data are not required provided that resolution is sufficient relative to the distance migrated. Thus, satellite platform terminal transmitters that estimate locations using the Doppler Shift could be used to model migration chronology [44]. Additionally, emerging technologies also offer increased opportunities to quantify migration chronology in small-bodied species. For example, a small radar transponder may be attached to an individual to enable tracking by a national network of weather radars, providing a practical method to track hundreds of birds each year without the cost or body size limitations of satellite transmitters [44], [45].

Enhanced information on migration chronology of waterfowl populations would allow agencies and organizations to efficiently allocate resources for conservation. Management activities could be targeted to specific time periods based on real-time data from a long-term monitoring program, but the specific method used to quantify migration chronology may affect inferences from monitoring efforts and the timing of other conservation activities. For example, a geopolitical approach could aid conservation efforts across broad spatial scales for the benefit of target and non-target species within a political jurisdiction. Regardless, waterfowl conservation efforts could be improved with a long-term migration monitoring program to document shifts in migration chronology due to climate change and yearly variation in environmental variables (e.g. precipitation, temperature) [46].

Numerous techniques have been used to quantify the timing and duration of migration in waterfowl, including radar [19] and radio-telemetry [20]. Although the study of migration chronology has been historically limited spatially and temporally, new technologies and methods have enabled researchers to develop quantitative techniques to define migration at the individual level [26], [27], [43]. Additionally, new movement models may be developed [*sensu* 27] and compared to established models. As a result, displacement metrics (NSD and ND) are simple yet informative measures that can be used to further develop quantitative approaches to studying avian migration ecology.

## Supporting Information

Table S1 **Variables and statistical tests for analysis of variance models that examined migration chronology, including initiation date, midpoint, and termination date, for a sample of 19 midcontinent mallards (** ***Anas platyrhynchos*** **) from 2010–2012.**
(DOCX)

Table S2 **Parameter estimates, standard errors, and associated test statistics for nonlinear mixed-effects models that quantified migration chronology of midcontinent mallards from 2010–2011.** Distance units are in km, midpoint units are in days since Day 0, and scale units are in days. Std Dev represents standard deviations for the random-effects in the model, which were included for all parameters except scale.
(DOCX)
