# Supplementary material for: Quantitative and Qualitative Approaches to Identifying Migration Chronology in a Continental Migrant
Source: PLoS One. 2013 Oct 9;8(10):e75673. doi: 10.1371/journal.pone.0075673 (PMC3794004; doi:10.1371/journal.pone.0075673)
Supplement: Table S1 — Variables and statistical tests for analysis of variance models that examined migration chronology, including initiation date, midpoint, and termination date, for a sample of 19 midcontinent mallards ( Anas platyrhynchos ) from 2010–2012. (DOCX) [file pone.0075673.s001.docx]

**Table S1. Variables and statistical tests for analysis of variance models that examined migration chronology, including initiation date, midpoint, and termination date, for a sample of 19 midcontinent mallards (*Anas platyrhynchos*) from 2010 – 2012.**

|  |  |  |  |  |  | 95% Confidence Intervals^3^ | |
| --- | --- | --- | --- | --- | --- | --- | --- |
| Season | Parameter | Variable | *F* | *p* | ***ω****^2^* | Lower | Upper |
| Autumn^1^ | Initiation | Method | 0.00 | 0.95 | -0.03 | 0.00 | 0.04 |
|  |  | Cohort | 2.48 | 0.13 | 0.05 | 0.00 | 0.30 |
|  |  | Year | 0.05 | 0.82 | -0.03 | 0.00 | 0.12 |
|  | Midpoint | Method | 0.00 | 0.99 | -0.03 | 0.00 | 0.00 |
|  |  | Cohort | 6.19 | 0.02 | 0.16 | 0.00 | 0.42 |
|  |  | Year | 2.25 | 0.15 | 0.04 | 0.00 | 0.29 |
|  | Termination | Method | 0.01 | 0.93 | -0.03 | 0.00 | 0.06 |
|  |  | Cohort | 2.61 | 0.12 | 0.05 | 0.00 | 0.31 |
|  |  | Year | 4.33 | 0.05 | 0.11 | 0.00 | 0.38 |
| Spring^2^ | Initiation | Method | 0.82 | 0.37 | -0.01 | 0.00 | 0.18 |
|  |  | Cohort | 0.07 | 0.79 | -0.03 | 0.00 | 0.11 |
|  |  | Year | 0.54 | 0.47 | -0.01 | 0.00 | 0.16 |
|  | Midpoint | Method | 0.13 | 0.72 | -0.02 | 0.00 | 0.12 |
|  |  | Cohort | 0.12 | 0.73 | -0.02 | 0.00 | 0.12 |
|  |  | Year | 2.26 | 0.14 | 0.03 | 0.00 | 0.25 |
|  | Termination | Method | 0.06 | 0.80 | -0.03 | 0.00 | 0.10 |
|  |  | Cohort | 0.10 | 0.75 | -0.02 | 0.00 | 0.11 |
|  |  | Year | 3.07 | 0.09 | 0.06 | 0.00 | 0.28 |

^1^*F* tests for autumn had 1 numerator and 25 denominator degrees of freedom

^2^*F* tests for spring had 1 numerator and 33 denominator degrees of freedom

^3^95% Confidence Intervals are for ***ω****^2^* estimates of effect size.
