# Supplementary material for: Quantitative and Qualitative Approaches to Identifying Migration Chronology in a Continental Migrant
Source: PLoS One. 2013 Oct 9;8(10):e75673. doi: 10.1371/journal.pone.0075673 (PMC3794004; doi:10.1371/journal.pone.0075673)
Supplement: Table S2 — Parameter estimates, standard errors, and associated test statistics for nonlinear mixed-effects models that quantified migration chronology of midcontinent mallards from 2010–2011. Distance units are in km, midpoint units are in days since Day 0, and scale units are in days. Std Dev represents standard deviations for the random-effects in the model, which were included for all parameters except scale. (DOCX) [file pone.0075673.s002.docx]

**Table S2. Parameter estimates, standard errors, and associated test statistics for nonlinear mixed-effects models that quantified migration chronology of midcontinent mallards from 2010 – 2011.** Distance units are in km, midpoint units are in days since Day 0, and scale units are in days. Std Dev represents standard deviations for the random-effects in the model, which were included for all parameters except scale.

| Cohort | Model | Day 0 | Parameter | Estimate | Std Error | *df* | *t* | *p* | Std Dev |
| --- | --- | --- | --- | --- | --- | --- | --- | --- | --- |
| SK | Single^1^ | 25 Sept 2010 | Distance | 1413.27 | 119.19 | 2056 | 11.86 | < 0.01 | 235.42 |
|  |  |  | Midpoint Autumn | 45.43 | 1.78 | 2056 | 25.59 | < 0.01 | 3.38 |
|  |  |  | Scale Autumn | 4.76 | 0.46 | 2056 | 10.30 | < 0.01 |  |
| SK | Double^2^ | 25 Sept 2010 | Distance | 2153.96 | 53.58 | 3692 | 40.20 | < 0.01 | 106.22 |
|  |  |  | Midpoint Autumn | 55.01 | 2.36 | 3692 | 23.33 | < 0.01 | 4.70 |
|  |  |  | Scale Autumn | 190.81 | 4.04 | 3692 | 47.23 | < 0.01 |  |
|  |  |  | Midpoint Spring | 5.60 | 0.15 | 3692 | 38.41 | < 0.01 | 8.06 |
|  |  |  | Scale Spring | 12.77 | 0.20 | 3692 | 63.38 | < 0.01 |  |
| AR | Single^3^ | 16 Feb 2011 | Distance | 1504.42 | 140.26 | 5103 | 10.73 | < 0.01 | 370.69 |
|  |  |  | Midpoint Spring | 40.92 | 7.01 | 5103 | 5.84 | < 0.01 | 18.52 |
|  |  |  | Scale Spring | 10.32 | 0.28 | 5103 | 37.01 | < 0.01 |  |
| AR | Double^4^ | 16 Feb 2011 | Distance | 2304.55 | 399.02 | 3221 | 5.78 | < 0.01 | 797.32 |
|  |  |  | Midpoint Spring | 42.04 | 6.22 | 3221 | 6.76 | < 0.01 | 12.42 |
|  |  |  | Scale Spring | 266.59 | 4.60 | 3221 | 57.97 | < 0.01 |  |
|  |  |  | Midpoint Autumn | 16.39 | 0.25 | 3221 | 65.26 | < 0.01 | 9.15 |
|  |  |  | Scale Autumn | 14.44 | 0.34 | 3221 | 42.24 | < 0.01 |  |

^1^Log-likelihood = -15543.49, AIC = 31100.98

^2^Log-likelihood = -24558.22, AIC = 49140.44

^3^Log-likelihood = -36056.40, AIC = 72126.80

^4^Log-likelihood = -21821.54, AIC = 43667.08
